# Root causes and fields of action to address unplanned hospitalisation in long-term care: a multiple case study with root-cause analysis

**DOI:** 10.1186/s12912-025-04206-2

**Published:** 2025-12-11

**Authors:** Nadine Janis Pohontsch, Tilman Alexander Huckle, Anne-Marei Jarchow, Katrin Balzer, Katharina Theodora Silies

**Affiliations:** 1 Department of General Practice and Primary Care, University Medical Center Hamburg-Eppendorf, Martinistr. 52, 20246 Hamburg, Germany; 2 Academy of the German Red Cross Schleswig-Holstein, Werner-von-Siemens-Straße 16, 24568 Kaltenkirchen, Germany; 3 Institute for Social Medicine and Epidemiology, Nursing Research Unit, University of Lübeck, Ratzeburger Allee 160, 23562 Lübeck, Germany

**Keywords:** Nurse’s role, Root cause analysis, Professional competence, Qualitative research, Hospitalisation, Long-term care, Nurses improving care for health system elders, Nursing homes

## Abstract

**Background:**

Being of older age is associated with chronic conditions and multimorbidity, which may lead to complex care needs of the persons affected. Residents in care homes are more often hospitalised than older adults living at home, and hospitalisations are often unplanned. It not sufficiently clear, how processes preceding unplanned hospitalisations are characterised, who is involved, what their decisions and competencies are, and what is needed to prevent such situations. We aimed to identify major causal factors for unplanned hospitalisations, potential fields of actions and goals for care.

**Methods:**

We conducted a multiple case study including a root-cause analysis based on cases of residents in care homes with unplanned acute care incidents as key events. We extracted data from 5 care home residents’ records and interviewed 18 involved parties (health professionals, residents, relatives) in two facilities. We analysed data from 8 weeks preceding the key event on case base with event flow diagrams and qualitative content analysis of case-related interviews. Based on causal factors for gaps in care we derived fields of action and goals for care.

**Results:**

Across cases, major causal factors were insufficient management of chronic diseases, disrupted interprofessional collaboration processes, and lack of individualised protocol application. We developed 31 fields of action with goals for care and clustered these into 7 overarching meta-themes: geriatric nursing processes; interprofessional collaboration; empowerment of residents; health promotion and prevention; communication processes; management; values, standards and guidelines.

**Conclusions:**

Caring for residents with complex care needs in long-term care requires nurses with expanded skills in clinical nursing, cooperation and communication. The results of this study contribute to the development of a new role profile for these nurses in German long-term care homes and specify relevant fields of action and goals for care.

**Patient or public contribution:**

An advisory board including resident representatives discussed and supported the project in repeated consultation meetings throughout the research process.

**Trial registration:**

The study was positively evaluated by the ethics committee of the University of Lübeck (file number 21–301) and registered prospectively at the German register for clinical trials (DRKS00025773).

**Clinical trial registration:**

German Register for clinical trials: DRKS00025773. Registered prospectively on 20th of August 2021.

**Supplementary Information:**

The online version contains supplementary material available at 10.1186/s12912-025-04206-2.

## Background

Being of older age or aging is often associated with a rising risk for chronic disease burden and multi-morbidities, disabilities and care dependencies [1]. Multimorbidity can be defined as being affected by three or more chronic conditions [2] It is prevalent with 25.5% in the age group 42–60 years, with 51.9% in the age group 61–80 years and with 67.3% in adults over 80 years of age [2]. Acute as well as chronic conditions and their consequences may imply increasing support needs of the persons affected. Monitoring symptoms of chronic diseases, cognitive impairment, an overall high level of dependency and care requirements at the end of life lead to increasingly complex care needs of the persons affected [3, 4]. Many older adults living in long-term care homes live with these conditions and frequently receive acute medical care. A cross-sectional study of 44 German nursing homes showed that 45.8% of residents aged 60 years or older were hospitalised at least once in the preceding 12 months with a mean of 1.6 hospital admissions in this group [5]. While hospitalisation is frequently necessary, a systematic review of 77 articles found that 4–55% of these hospitalisations were deemed avoidable [6]. The authors explicitly point out that the definition of appropriateness of hospitalisation is not uniform in the included studies and needs further investigation. Emergency department staff (physicians and nurses) in Belgium perceived most emergency calls for nursing home residents as avoidable if better primary care was provided, referring to more involvement of general practitioners (GP), more and better educated nursing staff and readily available and appropriate advance directives [7]. Decisions on hospitalisations are influenced by unclear expectations of the care providers, lack of staff, fear to mandate, and fear of exceeding their own scope of practice and responsibilities. Inadequate access to multidisciplinary ambulatory medical care at the care home and inadequate communication with other decision-makers can contribute to a decision in favour of treatment in hospital although this might be avoidable [8]. The main responsibility for deciding about the initiation of emergency medical services in potentially critical (health) situations of residents in care homes lies with nursing professionals. In Germany, the largest group of nursing staff providing direct care in long-term care are nurses with a three-year vocational training (43%) and nurse assistants with a one-year vocational training (13%). A substantial amount of 26% are staff without primary qualification related to nursing who have undergone a basic training to provide fundamental care [9]. Responsibility for the organisation of this skill mix in nursing staff lies with the nurse manager and head nurses leading wards or living areas. Other health care professionals in nursing homes are physiotherapists, logopaedics or occupational therapists who can be employed by the nursing home, but mostly are externally based and provide therapy for individual residents based on prescriptions by a GP or specialist physician. Primary medical care and specialised medical care is mostly provided by physicians of resident’s choice. Nursing homes rarely have on-site physicians which means that nurses have to coordinate medical care with many different GPs and specialists [10]. GPs usually cannot be contacted out of hours and often are not available for spontaneous visits to the long-term care home to evaluate residents’ health status in addition to planned visits [11]. In German long-term care, the proportion of nurses with academic training was less than 1% in 2021. Advanced nursing roles such as nurse practitioners or advanced practice nurses who autonomously provide primary care are not yet implemented. In situations with acute deterioration of a resident’s health, nursing staff therefore call for emergency services and / or initiate hospital admission. In a comprehensive systematic review Chambers et al. included 124 publications on interventions to reduce unplanned hospital admissions from nursing homes [12]. They found that integrated care and quality improvement programmes appeared to reduce unplanned admissions, but that interventions are complex and require adaptation to the local context. It is therefore necessary to know how processes that precede these unplanned hospitalisations in German long-term care homes are characterized: what was the development in resident’s health condition, who is involved, what are their decisions and competencies, and what is needed to prevent such situations. We aimed to address this knowledge gap and develop suitable strategies to prevent critical situations of residents in care homes, assuming that expanded nursing role profiles may be one way to improve person-centred care and reduce unnecessary hospitalisation. We understand person-centred care as care guided by individuals’ life goals and preferences which can improve the quality of health care delivery. Main elements of person-centred care are an individualised, goal-oriented care plan, collaboration in an interprofessional team and continual information sharing [13]. Expanded competencies can be described as activities that are not usually part of nursing roles in the setting, that formerly belonged to other professions’ roles, that involve new procedures or technologies or that require a higher degree of autonomy [14].

In line with current regulation of the entry qualification to the nursing profession in Germany, the majority of nurses have a three-year vocational training with diploma (in the following referred to as registered nurses or RN). The initial training is often followed by comprehensive continuous training in specialised fields such as intensive care, oncology nursing or palliative care. To meet increasing care complexities and demands upon care professionals, pilot projects for undergraduate academic nursing training were established in Germany around 2003/2004. With the introduction of the nursing professions act (PflBG) in 2020, regular professional approval based on a primary qualifying nursing degree on bachelor level is now possible. Basic nursing care is a primary duty of bachelor graduates, but it has been complemented by process responsibility in complex and highly complex nursing situations and cases [15].

Still, in Germany, there is a lack of practical definitions of specific practice areas and competence-oriented differentiation of task and responsibility frameworks for bachelor qualified nurses, especially in the field of long-term care. In particular, nursing care for residents with complex care needs comprises a scope of practice that matches competencies of nurses with this qualification level [16]. Examples of this are autonomous scientific analysis and problem-solving in complex care situations.

The multiple case study described in this paper was part of the Expand Care-project (Expanded nursing competencies to improve person-centred care for nursing home residents with complex health needs) [17]. The project consisted of two phases: a development phase and a pilot phase. The aim was to develop and evaluate an intervention to promote the qualification-based integration of registered nurses with Bachelor’s degree to improve care for older adults residing in care homes. The multiple case study contributed to the intervention development phase by exploring the need for a new model of care [18]. The overall results of the intervention development are reported elsewhere [19].

## Methods

### Aim and research question

The aim of the multiple case study was to contribute to the development of a new nursing role profile for bachelor qualified nurses in long-term care by identifying priority problems and goals of care for residents with complex needs in nursing homes. We addressed two research questions: (1) What characterises care processes before acute care utilisation (hospitalisations or emergency services) of residents with complex care needs in long-term care, especially in situations when nurses perceive decision-making as difficult? (2) Which structures, processes and outcomes indicate breaks or gaps in the continuity of care and result in unplanned hospitalisation?

### Design

We conducted a multiple case study [20]. A case study is a systematic examination of a phenomenon in its real life context and especially useful when phenomenon and context cannot be clearly separated from each other [20]. The phenomena of interest in this case study were context and processes around unplanned hospitalisation and other acute care service demands. Therefore, cases should contain at least one key event such as unplanned hospitalisation or other acute care service utilisation. To ensure that the findings reach a sufficient depth and breadth we studied multiple cases with different reasons for unplanned hospitalisations. We conducted the study in two long-term care homes incorporating qualitative interviews, a document analysis, and a root cause analysis (RCA [21]). Root cause analysis is designed as a method that can support the identification of not only what and how an event occurred but also why it happened [20], and it has been employed before to examine potentially preventable hospitalisation of residents living in care homes [22].

### Case selection

We aimed to include male and female residents with moderate and severe care dependencies to reach a maximum of variation in the information gathered [23]. Our recruitment aim was to include at least four cases in the study, with four to five interviews per case (with residents, relatives, surrogates, nursing and other professionals).

Potential critical cases were identified by nurse managers or head nurses [24]. We took care to ensure a heterogeneous sample in terms of cognitive status of the resident and type of the key event. Residents were excluded if they were in respite care, had been living in the care home for less than 2 months, were not currently living in the care home, or were likely to die within the next 3 months.

Residents were eligible for inclusion if they fulfilled one of the following conditions:

- The resident had had two or more unplanned contacts with acute medical services in the last 8 weeks (e.g. hospital admission or on-call contact or outpatient treatment in the emergency room).
- The resident had had one unplanned contact with acute medical services in the last 8 weeks (e.g. hospital admission or on-call contact or outpatient treatment in the emergency room) and, additionally, the nursing manager or head nurse identified the case as particularly relevant due to the complexity of care (clinical judgement).

Registered nurses, nursing assistants, general practitioners or other health care professionals were eligible for interviews if they had been involved in care processes with the resident on a regular basis before the key event. Relatives or surrogates, or further persons relevant to residents were eligible for interviews, if they had been the residents’ primary contact in the last two months before the key event.

### Study setting, recruitment and inclusion

We invited long-term care homes in the south of the federal state of Schleswig-Holstein in Northern Germany. Facilities had already worked with the University of Lübeck as a research or education partner or had already confirmed their support for the Expand-Care project. Two long-term care homes agreed and offered their support in the recruitment of residents and data collection. The recruitment of care homes took place in August 2021. Nursing managers informed residents meeting the inclusion criteria and legal representatives, if applicable, verbally and in writing about the study and asked for their consent to participate. The written consent referred to the inspection of the residents’ documentation in the long-term care home by the research team, the participation in one and, if necessary, further interviews, and the consent to contact and interview other agents involved. In the case of residents with legal representation or care, we obtained consent from their legal representatives.

After residents’ (or legal representatives’) written consent, nursing managers informed additional involved agents meeting the inclusion criteria (in writing/per telephone) about the study and the resident’s consent, and invited them to participate. We determined the relevance of these involved agents for the study on the basis of the documentation or in the course of preceding interviews [emergent sampling [24]. We provided study information verbally and in writing, and involved agents gave written informed consent to take part in the study. The recruitment of cases was completed in September 2021.

### Data collection

After consent was given, we extracted data from residents’ electronic records (KS, TH, AMJ) and conducted interviews with the residents and other involved agents.

#### Data extraction from residents’ electronic records

We created a data extraction sheet to collect relevant data in a standardised manner from residents’ electronic care home records and from paper-based documentation where applicable. Extracted data items are depicted in Table 1. Relevant data items were identified through previous studies [e.g [25]], and discussed and reviewed within the research team.

**Table 1:** Data items extracted from residents’ records

Data items extracted from care home residents’ records	
• Sociodemographic data • Diagnoses • Degree of care dependency • Current wounds or infections • Continuous and on-demand medication • Medical and nursing aids, supplies or drains • Care reports, release letters from hospitals and supplementary records (fall records, fluid intake monitoring, faxes or emails with other care providers) for the last two months preceding the key event • Consultations in the last two months preceding the key event (date, type of consultation, occasions and processes leading to the consultation, initiator of the consultation), including o planned doctor visits, hospital admissions o unplanned doctor visits, on-call/emergency doctor visits, hospital admissions • Specifications in documents for advance directives, health care proxy, legal care, living will, if applicable.	

#### Interview guides and interview conduction

Semi-structured interview guides [26, 27] for all groups of involved agents were developed by NP and discussed and reviewed within the research team (KS, AMJ, KB, NP). Additionally, standardised forms for the collection of sociodemographic data from the interviewed persons were used, in order to describe the study population (age, gender, relationship to resident, professional occupation and qualification). The complete semi-structured interview guides can be found in supplement A. For an overview, main interview topics are depicted in Table 2. All interviewers (KS and TH) were familiar with conducting qualitative interviews and had no previous relation to the persons interviewed. Interviews took place in private either in the long-term care facility or via telephone. The interviews were digitally audio-recorded and subsequently transcribed by the research team. Audio data was deleted after transcription. The data collection ended in October 2021.

**Table 2:** Main topics of interview guidelines per group of involved agents

	Resident	Relatives	RN & NA	Nurse Manager	GP & THER
Description of daily life/typical activities in the care home	X	X	X	X	
Description of current health status and care dependency of the resident	X	X	X		X
Description of typical contacts and communication with involved agents (e.g. nurses, residents, GP) in the care home	X	X			X
Communication with relatives and legal representatives	X			X	
Processes when resident is feeling unwell	X	X	X	X	X
Evaluation of the key event or situation	X	X	X		X
Competencies, interventions or resources missing in the care home	X	X			
Current skill-mix in the care home				X	
Improvement needs for care within the care home			X	X	
Improvement needs in the interprofessional network				X	
Recently implemented changes in care processes				X	
Processes for interprofessional communication and collaboration in the care home			X	X	
Advance care planning in the care home				X	

RN: Registered nurses; NA: Nursing assistant; GP: General practitioner; THER: Therapist

### Data analysis

RCA was used in order to identify the underlying causes for a serious event or critical problem that occurred [21]. This methodology assumes that a critical event does not occur due to one single fault or mistake, but rather that there are multiple causes that added up and preceded the event. Rooney and van den Heuvel [21] describe four steps of the root cause analysis: data collection, causal factor charting, root cause identification and recommendation. Table 3 shows how we implemented every step of the method in our multiple case study.

**Table 3:** Root cause analysis steps and implementation of the steps in the multiple case study

Step according to Rooney & van den Heuvel (2004)		Implementation in the multiple case study
Data collection	*Collection of available data in association with the key problem*	• Data extraction from residents’ records • Interviews with relevant involved agents
***Case-based analysis***		
Causal factor charting	*Structured diagramming with description and identification of the conditions/events/persons associated with the key problem*	• Event flow diagram: events, communication and processes leading up to the key event or situation chronologically ordered per case. • Linking of data from documentation and interviews in the event flow diagram. • Identification of causal factors for deficits in care. *Example: Relative noticed oedema and dark urine preceding resident’s heart attack.*
Root cause identification	*Identification and mapping of root causes and primary difficulty sources*	• Asking questions to discuss causal factors and derive more general potential fields of action. *Example: Are nurses able to recognise exacerbation symptoms of chronic heart failure?* • Developing fields of action linked to generalising questions per case. *Example: Symptom control in chronic diseases*
***Cross-case analysis***		
Recommendation	*Creating achievable recommendations to reduce or eliminate the problem*	• Consolidating fields of action across cases and clustering them into meta-themes • Fields of action contain goals for care and potential nursing task to address these goals. *Example: Exacerbation of chronic diseases is detected early – Implementation of in-house protocols for the assessment of chronic diseases.*

### Case-based analysis

First, we (KS and TH) organised data collected from residents’ records according to an event flow diagram to structure each case. Subsequently, we plotted events, processes, interventions and communication that preceded the key event and were relevant to the case into this event flow diagram. Interview data – descriptions of relevant events and processes – were arranged according to the event flow diagram and thus supported and expanded document-based data through interviewees’ perspectives.

Through qualitative content analysis of the clustered interview data [28], we inductively derived causal factors for breaks or gaps in care. These gaps / breaks in care processes could be for example (a) missing or incomplete information or communication, (b) unmet health needs of residents, or (c) missed opportunities for prevention or the timely initiation of interventions in long-term care. Regarding unplanned emergency medical services, a gap or break in care could also mean an indication of something that could have prevented hospitalisation or contact with the emergency services.

We proceeded in each case in a similar way. Based on the causal factors in each case, we formulated generalising questions. These questions (rather than pointing to concrete events as mistakes) provided a neutral prompt to open the perspective from the specific case constellation towards broader potential fields of action, and supported the formulation of goals of care connected to these fields. The final result for each case was an event flow diagram, causal factors for gaps in care present in the case, and formulated generalising questions indicating broader fields of actions. The latter describe specific areas where nurses or teams can take deliberate actions to achieve goals or create change, for example: “Participation of relatives in the care process”. Box 1 and Table 5 in the results section illustrate the output of this process.

**Box 1:** Exemplary case summary

Case 1: Resident living with dementia and repeated falls
The key event in this case was a hospitalisation after the resident fell and acquired a minor head wound. The resident (“they” or R1 in the following) lived with dementia, showed a wandering behaviour with a history of repeated falls and fall related injuries. R1 had received antipsychotic medication since entry into the facility several years ago. After an initial period of higher dosage, medication had been reduced. R1 was provided with a helmet and hip protectors to prevent fall-related injuries but accepted these only intermittently. Additionally, R1 had a wristband alerting staff if they left the facility. Their spouse visited regularly and communicated with nurses in charge and the general practitioner (GP). The spouse initiated a medication review with the GP after observing R1 to appear dizzy. Regarding the decision to transfer R1 to the hospital after the fall, nursing staff referred to a general routine to hospitalise any resident after a fall if injuries to the head occurred. Overall, R1’s spouse was very satisfied with the care provided by staff and felt that they reacted well if the spouse suggested improvements for R1’s care.

For this analysis we initially worked paper-based and used MAXQDA Standard V.20 software [29] to document case-based results for the integrated analysis of all cases.

### Cross case analysis

After the case-based analyses, we discussed and reviewed in the interprofessional research team the fields of action that we derived from the case-specific generalising questions and formulated goals for care. We appointed a range of potential nursing activities to the fields of action to address each goal. We applied fields of action already identified in one case to further cases if this was supported by data in an iterative process. Through this iterative development and application process, we consolidated fields of action and associated nursing activities across all cases. We finally clustered all fields of action into meta-themes. These meta-themes can be described as overarching areas in which expanded competencies of nurses in long-term care can be located.

### Rigor and reflexivity

We employed several measures to ensure the rigour of the study [30]. We created a data extraction sheet based on previous research [25] to standardise data extraction from nursing documentation and used a standardised form to assess sociodemographic data. We used a semi-structured guide (created by the interprofessional research team of the Expand-Care project) to ensure that all relevant topics were discussed with each interviewee while allowing for openness to expressions of personal views [26]. Trained and experienced interviewers conducted the interviews. Two researchers conducted the qualitative data analysis together and continuously discussed results with the interprofessional research team. Team members had professional qualifications in nursing, care for older adults, quality management, psychology, public health and primary care. We collected and analysed data from different sources (interviews, residents’ records) to gain a holistic picture and detailed understanding of each case under study. Using an event flow diagram and qualitative content analysis [28] for the interview data, we clustered data from different sources in themes, thus comparing and contrasting all perspectives in a case. We analysed cases iteratively and assumed data saturation when we did not find new fields of action in the last two cases we analysed. The reporting of the manuscript followed the COREQ guideline (Consolidated Criteria for Reporting Qualitative Research) [31].

### Ethical considerations

Participation in the study was voluntary. All participants received written information about the aims of the study, the study procedure, the voluntary nature of participation, the storage and use of data and the data protection concept. The study was positively evaluated by the ethics committee of the University of Lübeck (file number 21–301) and registered prospectively (DRKS00025773).

During recruitment, all participating residents (cases) received an ID (pseudonym) in a separate identification list stored under protection at the university. The ID was used in all further study documents. All data belonging to a case were linked by means of this ID number. The interviews were pseudonymised and all identifying details were removed during transcription. Participants were offered 25 Euro as compensation for the interviews.

## Findings

### Characteristics of participants

Two long-term care homes participated in the multiple case study. One facility was privately owned with more than 150 beds. The other one with fewer than 100 beds was part of a larger non-profit organisation. One facility was located in a rural, the other in an urban area. Five cases were included in the multiple case study, and we reached our recruitment aim on the level of cases. We conducted interviews with 18 involved agents, and missed our recruitment aim on this level. The interviews lasted between 10 and 60 min.

The recruitment of relevant participants proved difficult with general practitioners who declined participation due to time constraints in all but one cases. Two residents were unable to actively participate in interviews due to their living with severe dementia. An overview of the interviews conducted per case is given in Table 4. Some interviewees (a registered nurse, the nurse managers and the physiotherapist) were involved in more than one case, but reviewed all respective cases in one interview per involved agent (indicated by an asterisk in Table 4). We report the characteristics of participants and cases in a summarised manner to strengthen the anonymisation.

**Table 4:** Interview participants

Case	Long-term care home 1			Long-term care home 2	
	1	2	3	4	5
Resident	*n.p.*	Interview	Interview	Interview	*n.p.*
Relatives	Interview	n.a.	Interview	Interview	Interview
RN	Interview	Interview*		Interview	Interview
NA	n.i.	Interview	n.i.	Interview	Interview
NM	Interview*			Interview*	
GP	n.a.	n.a.	Interview	n.a.	n.a.
THER	n.i.	n.i.	n.i.	Interview	

RN: Registered nurse; NA: nursing assistant; NM: nursing manager; GP: general practitioner; THER therapist; n.a.: not available; n.i.: not involved in case; n.p.: residents living with dementia, active participation was not possible. *Some interviewees (RN, NM, TP) were involved in more than one case and cases were reviewed together in one interview per agent

#### Residents (*n* = 5 cases, *n* = 3 interviews)

Five cases (residents; 4 female and 1 male) of moderate or high levels of care dependency were included. In all cases key events were hospitalisations, due to: falls, unclear health deterioration, heart failure or anaemia. All residents had multiple diagnoses, among these musculoskeletal diseases, heart diseases, incontinence, and dementia.

#### Relatives (*n* = 4)

Participating relatives all acted as surrogates for their family member. Contact frequency differed from monthly visits or phone calls to up to three weekly visits. Their involvement in care processes varied and comprised mostly social activities but in one case (Case 4) also regular communication with the GP.

#### Registered nurses (*n* = 4)

The nursing professionals were experienced with at least 5 years of work and had additional qualifications such as team leadership. They were responsible especially for medication administration, creation of care plans and interprofessional communication.

#### Nursing assistants (*n* = 3)

Nursing assistants had varied work experience (1 to 12 years) and qualifications (one-year nursing assistants’ education or less). They were responsible for basic daily care including body hygiene, mobilisation and dressing.

#### Nursing managers (*n* = 2)

Nursing managers were experienced (more than 5 years of work experience and at least two years in a leading position). They were responsible for the organisation and the quality assurance of care within their long-term care home and had several further formal qualifications.

#### General practitioner (*n* = 1)

One GP participated in the multiple case study. The GP was responsible for the medical treatment of a number of residents as well as the communication with nursing staff of long-term care homes.

#### Physical therapist (*n* = 1)

One self-employed physical therapist participated in the multiple case study. The physical therapist had more than 10 years of experience as a therapist and equally in the collaboration and communication with long-term care homes.

### Results of the case-based analysis

In this section we report the results of the root cause analysis on case level. To illustrate the process of the analysis, we first show a case summary and causal factors we found in this case. We then illustrate the next analysis step for the example case 1, with generalising questions and associated fields of action. Case summaries and full information on results of all cases are presented in supplement A. To veil participants’ gender, we use the pronouns “they/their/them” instead of “he/his/him” or “she/her”.

From the case presented in Box 1 we identified the following causal factors for gaps in care:

- A relative initiated the medication review instead of the nurses.
- Not a nurse, but a relative observed dizziness and interpreted this as related to antipsychotic medication.
- Medical treatment was not adjusted after key events.
- Nurses took measures to prevent fall-related injuries but not to prevent falls.
- Nurses followed seemingly implicit rules on how to decide on hospitalisation (“if the head is involved, they always go to hospital”), even if the assessment of the resident’s actual condition did not show any symptoms.

We proceeded in each case in a similar way. Table 5 shows the results of this process for case 1, results for cases 2 to 5 are shown in supplement A.

**Table 5:** Generalising questions and associated fields of action in case 1

Questions	Fields of action
• What is the role of nurses in the management of neuroleptic / antipsychotic medication?	• Involvement of nurses in management and medical care of chronic diseases
• Why does the relative initiate a review of neuroleptics/antipsychotic medication with the GP and not the nurse?	• Participation of relatives in the care process
• What do nurses understand by preventive actions regarding falls in long-term care?	• Active initiation of preventive measures
• How does communication between GPs and specialists take place regarding residents in long-term care, and which role do other involved agents (nurses, relatives) play?	• Communication and collaboration with general practitioners and specialists
• Do values, implicit and explicit facility rules lead to more hospital admissions than necessary? • What is the role of previous failure/success in critical situations in care in decision-making processes? • Which explicit or implicit standards exist for information flow, scope of practice and decision-making in long-term care?	• Handling rules and protocols • Shaping learning processes in the facility • Organising teams with mixed skills levels

### Results of the multiple case analysis

We clustered fields of action into seven meta-themes. Table 6 shows an overview of these meta-themes with the fields of action each meta-theme comprises. The overview indicates in which case fields of action were first developed (marked by “X”) and in which cases they were further applied (marked by “O”). We analysed case 4 first, followed by case 3, 1, 5, and lastly 2. We assumed data saturation when we did not find new fields of action in the last two cases we analysed.

**Table 6:** Overview of fields of action clustered into meta-themes and cases

Meta-theme	Fields of Action	1	2	3	4	5
1. Geriatric nursing care	1.1. Symptom control in chronic diseases	O		O	X	
	1.2. Involvement of nurses in medical care				X	
	1.3. Understanding health as a life course process				X	O
	1.4. Case-management				X	O
	1.5. Evaluation of the care situation	O	O	O	X	O
	1.6. Reaction to nursing assessments	O		O	X	O
	1.7. Quality of care based on current scientific standards	O			X	
	1.8. Nursing support in the therapy of chronic diseases	X			O	O
2. Interprofessional collaboration	2.1. Multi-professional checklist	O			X	O
	2.2. Nurses competence for prescription of defined medical services				X	O
	2.3. Recognising and considering concerns of residents regarding hospitals		O		X	O
	2.4. Maintaining a trans-sectoral care network	O		X		O
	2.5. Medication after hospitalisation			X		O
3. Empowerment of residents	3.1. Prioritisation of care needs		O		X	O
	3.2. Enabling empowerment and participation		O		X	
	3.3. Advance Care Planning		O	X		
4. Health promotion and prevention	4.1. Strategies for health promotion				X	
	4.2. Active prevention	X				
5. Communication processes	5.1. Participation of relatives	O		O	X	
	5.2. Relatives as a resource in care processes	O			X	
	5.3. Nurses as advocates of residents		O	X	O	
	5.4. Communication with relatives and residents	X	O	O	O	O
	5.5. Communication with general practitioners and specialists	X	O	O		
	5.6. Communication in teams with mixed skills levels in the facility	O	O			
6. Management	6.1. Management of residents’ approved care level			X		
	6.2. Management of medical and care devices	O		X		
	6.3. Psychosocial management of residents moving into the facility			X		
7. Values, standards and guidelines	7.1. Handling rules and protocols	X			O	O
	7.3. Protocols as guidance for decision-making	X			O	O
	7.5. Shaping learning processes in facility	X				
	7.7. Organising teams with mixed skills levels	X	O			

X: Field of action primarily derived from the case; O: Field of action derived in a previous case and deductively associated to case

For each field of action, we formulated goals of care and potential nursing activities or interventions based on the causal factors identified and the expertise in the multiprofessional research team. In the following, we present each meta-theme with one example field of action to illustrate this.

1. *Geriatric nursing care* refers to the management of chronic and geriatric diseases, especially regarding geriatric assessments, detection of health deterioration, re-evaluation of care plans and support of medical therapy.
  Example: Field of action *1.1 Symptom control in chronic diseases* aims at the early detection of exacerbation of chronic diseases. Potential nursing activities to achieve this goal are the implementation of local / in-house protocols for assessment tools, carrying out these assessments and ensuring reaction in a timely manner.

2. *Interprofessional collaboration* refers to clear and comprehensive communication including all involved agents’ perspectives. Especially in transitions between care settings it is necessary to secure continuity in care.
  Example: Field of action *2.4 To maintain a trans-sectoral care network* aims at ensuring timely specialist medical care. Potential nursing activities are to agree on structured communication pathways with medical practices or to initiate case conferences by telephone or video with residents / relatives and physicians for complex cases.

3. *Empowerment of residents* means to accept residents’ wishes as primary guiding value for the prioritisation of care.
  Example: Field of action *3.2 Enabling empowerment and participation* aims to ensure that residents can make informed decisions. Potential nursing activities are to inform residents about the results of nursing assessments, benefits and risks of interventions and to initiate nursing visits and case discussions involving relatives or confidants on a regular basis.

4. *Health promotion and prevention* refers to nurses actively incorporating these topics in long-term care
  Example: Field of action *4.1 Strategies for health promotion* aims at informing residents about opportunities to take health promoting measures and support them in setting health goals. Potential nursing activities to reach this aim are to determine individual health values and realistic goals in a structured dialogue with residents and to identify and plan measures to support individual health behaviour as well as to adapt the environment and guide implementation.

5. *Communication processes* refers to the role of nurses in communication with all agents involved in residents’ care.
  Example: *Field of action 5.1 Participation of relatives* aims at establishing trusting communication with relatives so that information is not only passed on but a joint discussion and assessment is developed. Potential nursing activities are to conduct case conferences in which relatives are included and to actively seek the assessment of relatives as part of the care planning and the care process.

6. *Management* refers to coordinating and advancing different aspects of residents’ care situation and securing adequate means for care.
  Example: Field of action *6.3 Psychosocial management of residents moving into the facility* aims at supporting residents to adapt to their new home and surroundings when they move into the facility. Potential nursing activities are to carefully observe residents’ psychosocial conditions especially in the first phase after moving in and arranging an admission process that takes their needs for (psycho)social activities into account.

7. *Values*,* standards and guidelines* refers to explicit and implicit rules that are established in care homes and how they are applied to provide care according to individual residents’ needs.
  Example: Field of action 7.1 *Handling rules and protocols* aims at a balanced use of this guidance (securing quality of care processes while at the same time providing person-centred care). Potential nursing activities are to identify current implicit and explicit rules and standards of the facility, to develop criteria to measure outcomes of protocols, to regularly evaluate the use of protocols and to update protocols based on current evidence.

A full list of all fields of action with goals of care and nursing activities / interventions per meta-theme is provided in supplement A.

## Discussion

With this multiple case study, we analysed five cases of care home residents in need of unplanned acute medical care. Through a root cause analysis, we found a range of underlying causal factors that can lead to gaps in the care process and may contribute to the hospitalisation of residents in care homes. Prominent themes were a lack of continuity in the symptom control and management of chronic conditions, disrupted communication processes between involved agents, and values and standards, such as implicit rules for care decisions that could overrule residents’ care preferences.

Current international research mirrors the areas we identified in our study. For example, the management of chronic diseases includes polypharmacy and medication safety. A mixed-method review showed how specialised nurses can provide interventions to improve medication safety [32]. The importance of GPs providing medical care for residents in long-term care, especially in end of life care, has been shown [33]. Yet, both the workforce of GPs and specialists, as well as the nursing workforce are scarce, which emphasises the need for interprofessional collaboration and communication in nursing homes [34]. For situations when residents’ health status deteriorates, a need for standardized tools in deterioration management has been identified internationally, but currently there is little standardisation, and the evidence for these instruments is not yet clear [35]. Advance care planning in nursing homes is not yet fully implemented although it may support decision-making according to residents’ preferences, increase goal-concordant care, and reduce hospitalisation [36].

Our study showed that causal factors for gaps or breaks in the continuity of care are manifold and interrelated. Interventions to improve current care models will therefore have to target different fields of action simultaneously. Charmers and colleagues found that complex interventions centring on improving staff skills and processes of care and those centring on improving links between external health care providers and care homes showed the most success in reducing unplanned hospitalisations [12]. Importantly, the immense international differences between healthcare and long-term care systems have to be taken into account when implementing complex interventions to improve quality of care in this setting [37]. This underlines the importance of evaluating the specific context in which new care models are to be implemented, as we have done within this multiple case study.

This study was part of a larger research project, the Expand-Care study, in which we aimed to develop and pilot a new role profile for nurses with expanded competencies in long-term care. In the final phase of the root cause analysis in this multiple case study, we developed goals for care and drafted potential nursing activities or interventions to achieve these goals as part of this new role profile. Our results are 31 fields of action within seven areas: (1) Geriatric nursing processes; (2) Interprofessional collaboration; (3) Empowerment of residents; (4) Health promotion and prevention; (5) Communication processes; (6) Management; (7) Values, standards and guidelines. These areas should be addressed by nurses with expanded competencies in long-term care in order to improve person-centred care (PCC) and decrease avoidable hospitalisation of care home residents.

The core of person-centred care is that a person’s values and preferences guide all aspects of care, and that their realistic life goals are supported. The basis for person-centred care is the dynamic relationship between a person, their healthcare professionals and significant others. According to this widely acknowledged definition, PCC is characterised by eight elements that are essential to realise this definition [13]. Among these elements are for example an individualised care plan that is regularly reviewed, active coordination among all healthcare professionals and education and training for providers and other involved agents. Our results show that in long-term care there is a need for improved person-centred care with regard to these elements. We saw for example causal factors relating to insufficiently holistic and up-to-date care planning when addressing chronic diseases. Therefore, we defined fields of action such as “Reaction to nursing assessments” and “Quality of care based on current scientific standards” to improve geriatric nursing care. Also, disrupted collaboration and communication processes, as well as a lack of empowerment of residents and a judicious implementation of values, protocols and guidelines became apparent in our study. PCC elements to address these factors would be an individualised, goal-oriented care plan and an improved interprofessional team work [13]. A more recent umbrella review describes attributes of PCC and how theory can be translated into practice [38]. An important prerequisite to facilitate PCC is staff training and education but also the introduction and use of guidelines, tools and documentation of care plans. The latter refer to instruments that can enable awareness for a person’s needs and form care partnerships, and to tools that facilitate a structured individualised care plan. The American Association of Colleges of Nursing identifies person-centred care as the core purpose of nursing as a discipline and one of ten domains for nursing education. Competencies for person-centred care are described on an entry-level and an advanced-level nursing education, for example to engage with an individual in establishing a caring relationship, to integrate assessment skills in practice and to diagnose actual or potential health problems and needs, also reflecting fields of action we identified in our study [39].

The future distinguishing competencies of registered nurses with Bachelor’s degree in long-term care have been discussed in an international expert consensus study [40]. The experts identified 16 competencies in four areas: leadership and coaching, communication, evidence-based practice, and client assessment and geriatric expertise. The said expert consensus showed a strong focus on leadership and coaching as future competencies, interpreting this area broadly. It includes not only the coordination of a multidisciplinary team, but also expanded care planning that ensures necessary interventions are identified, conducted and their results evaluated. The lacking continuity of care for residents with chronic conditions we observed, confirms the need for this focus in long-term care. In our results, this is therefore reflected in the fields of action “Shaping learning processes in the facility” and “Organising teams with mixed skills levels”.

Internationally, a root cause analysis of hospital transfers from skilled nursing facilities by Ouslander et al. found that some hospital transfers could have been prevented and residents treated onsite if worsened health conditions had been recognised earlier [22]. In our study, resident assessment and geriatric nursing expertise also were of high importance. This may partly be due to the specific context of German long-term care where nurses have a limited scope of practice with regard to primary care interventions, although only few facilities have onsite physicians. Mostly, residents choose their general practitioner outside the facility, resulting in higher communication efforts with many different GPs for nurses. Usually, GPs cannot be contacted out of hours and often are not available for spontaneous visits to the long-term care home to evaluate residents’ health status in addition to planned visits [11].These barriers to communication and consultation with GPs can increase unplanned hospitalisations [41]. Additionally, in line with the expert consensus, our study showed a need for improved communication also with residents and relatives.

Reimbursement for long-term nursing care in Germany is determined by residents’ approved level of care – mainly assessed through needs in basic nursing care. But our study showed that the complexity of care is not only related to residents’ approved care levels, which were moderate to high in our residents, but also to chronic diseases and medical care needs. This means that increased nursing staff efforts to coordinate care are currently not sufficiently refinanced through the health insurance system in Germany.

Internationally, it has been shown that nurses with expanded competencies can increase the quality of care. While new roles for these nurses are often located at hospitals, it can be assumed that they might have even greater benefits in settings where a physician is not constantly available, as it is common in German long-term care [42]. A prerequisite for expanding roles with broader responsibilities is adequate education for the nurses. This comprises academic qualification on bachelor level supported by programmes for continuous qualification and specialisation for specific areas and settings. Our results show which areas need to be addressed in long-term care on this level. In countries with longer history of nurses in advanced roles, the benefit of nurse practitioners in long-term care homes has been shown [43]. To implement these advanced practice nursing roles, nurses with master and doctorate qualification are needed. In the context of our results, care processes and swift adaption of individualised care could be improved if care homes had for example onsite nurse practitioners with prescription autonomy. For German long-term care this is currently a vision for the future, but clarification of specialist roles in Europe is developing [44, 45].

### Strengths and limitations

To our knowledge this is the first multiple case study in Germany to address unplanned hospitalisations of care home residents in long-term care. We gained in-depth descriptions of care processes from different cases and multiple perspectives which allowed us to identify specific fields of action in a comprehensive manner and draft potential nursing activities and interventions to improve care. Thus, we created the knowledge base needed for the further development of an expanded nursing role profile on bachelor level to improve person-centred care in long-term care in Germany. Our results show that such a role and expanded nursing competencies to fulfil it are needed.

Our study has some limitations. Results rely mainly on subjective views of relevant involved agents. Those views could be biased by a social desirability, retrospective sensemaking and impression management [46]. By using different perspectives (residents, relatives, nursing staff etc.) and replenishing interview data with data from residents’ records we were able to depict cases comprehensively. Through the analysis of multiple cases, results from single cases were confirmed, which we interpret as a sign of data saturation [20], even though we did reach our recruitment aims only on the level of cases and not on the level of interviews with involved agents. The method of root cause analysis has been developed in the area of quality management, to explore and improve processes and to prevent recurrences of undesired events. We use the terms “root cause” and “causal factors” in our study as they are central to this method [21], but they do not imply “causality”. If the study aim had been to prove causality of factors determining hospitalisation, different study designs would have been needed. We could only recruit cases in two long-term care homes and some target groups, such as GPs, were hard to reach and are therefore underrepresented in the study. Still, based on the characteristics of the included care homes (representing varied size, area, and ownership) and cases (residents with varied age, diagnoses, and reasons for hospitalisation), and the depth of the analysis, we feel that we were able to get comprehensive insights. We therefore deem our results as transferable to the long-term care setting in Germany, and relevant to an aging population and to nursing professionals. However, we cannot deny that our results are specific to the German long-term care setting which may limit their transferability to other countries and settings. Further research should apply nationwide recruiting strategies or work with international comparisons.

### Implications for practice and research

Currently, less than 1% registered nurses with Bachelor’s degree are employed in long-term care in Germany. The majority of nursing staff has a 3-year vocational education, complemented by staff with one-year nursing education or less [47]. In the light of our results, we conclude that the competencies needed to provide nursing care for residents with complex needs as those included in our study, require the employment of bachelor educated nurses.

Our results contribute to the development of educational training for nurses with expanded competencies, the re-definition of skills and grade mix in long-term care teams and to the development of evaluation designs to measure the impact of these roles. The feasibility in practice and the effects of new nursing profiles on quality and person-centeredness of care need to be assessed through research methods that acknowledge their immanent complexity [12, 48]. Further, it has to be investigated, how many bachelor level nurses (and higher-level academic nurses) are needed in relation to vocationally trained nurses and nurse assistants. Current nursing staff may be ambivalent towards new roles that differentiate between vocationally and academically trained nurses in clinical practice in systems were nurses traditionally were trained at nursing schools and not at universities. Participatory research methods should therefore be employed to identify implementation barriers and design strategies accordingly [49, 50]. In the light of growing staff shortages in many countries, this may contribute to attracting highly qualified nurses to work in this setting. Lastly, the increasing availability of AI interventions to support nursing processes and nursing research will have to be considered when new care models are to be designed and evaluated [51].

## Conclusion

Caring for residents with complex care needs in long-term care requires nurses with expanded skills in clinical nursing, cooperation and communication. The results of this study contribute to the development of a new role profile for these nurses and specify relevant fields of action and goals for care. Next steps in our project are the definition of a new model of care based on our results and the planning and initiation, and evaluation of the new expanded nursing role and model of care. Further, our results can contribute to current international developments to clarify expanded roles, associated nursing interventions and responsibilities, and the required competencies.

## Supplementary Information

Below is the link to the electronic supplementary material.

Supplementary Material 1

## Data Availability

The data are not available due to privacy or ethical reasons.

## Declarations

### Permission to reproduce material from other sources

N.A.

## Abbreviations

GP: General practitioner
ID: Identifier, pseudonym
NA: Nurse assistant
NM: Nursing manager
PCC: Person-centred care
PflBG: [Pflegeberufegesetz] nursing professions act
RCA: Root cause analysis
RN: Registered nurse
R[x]: Resident [Nr.]
