# Supplementary material for: Root causes and fields of action to address unplanned hospitalisation in long-term care: a multiple case study with root-cause analysis
Source: BMC Nurs. 2025 Dec 11;25:44. doi: 10.1186/s12912-025-04206-2 (PMC12802160; doi:10.1186/s12912-025-04206-2)
Supplement: Supplementary file 1 — Supplementary Material 1 [file 12912_2025_4206_MOESM1_ESM.pdf]

Supplement A

Root causes and fields of action to address unplanned hospitalisation in long term care: a multiple case study with root-cause analysis

Pohontsch NJ, Huckle T, Jarchow A, Balzer K, Silies KT.

Table of contents

Directory of tables and figures 1
Section 1 – Supplementary material to methods 2
1. Topic guides 2
1.1 General practitioners 2
1.2 Nurses 4
1.3 Nursing manager 6
1.4 Relatives and significant others 8
1.5 Residents 10
Section 2 – Supplementary material to results 12
1. Case summaries 12
2. Case-based results: causal factors, generalising questions and fields of action 14
3. Fields of action, goals and interventions 20

Directory of tables and figures

Table 1 Topic guide for general practitioners 2
Table 2 Topic guide for nurses 4
Table 3 Topic guide for nursing managers 6
Table 4 Topic guide for relatives 8
Table 5 Topic guide for residents 10
Table 6 Causal factors, questions and fields of action case 1 14
Table 7 Causal factors, questions and fields of action case 2 15
Table 8 Causal factors, questions and fields of action case 3 16
Table 9 Causal factors, questions and fields of action case 4 17
Table 10 Causal factors, questions and fields of action case 5 19
Table 11: Fields of action, goals and interventions to eliminate gaps and breaks in care........ 20

Section 1 – Supplementary material to methods

1. Topic guides

1.1 General practitioners

Table 1 Topic guide for general practitioners

Hello. I am (name) and I work at (institute). We are meeting today because we want to talk about your patient Ms./Mr. (name). Ms./Mr. (name) was recently in hospital unplanned. I would like to talk to you about the medical care you provided to Ms./Mr. (name) and why she/he was in hospital. I have some general questions and some that relate to your patient Ms./Mr. (name).<br>All information you provide will of course be treated confidentially. Everything I learn during our conversation will remain between us and will only be used pseudonymously. This means that your name will not be published together with what you have said.<br>It is also important that there are no wrong or right answers here: I am interested in your opinion and perception. So please tell me everything that is on your mind.<br>If you agree, I would like to record the conversation on tape as discussed. If possible, please switch your cell phone / practice telephone to silent mode so that we are not disturbed.
Ms. / Mr. (name) is being cared for in a care home. Can you tell us what kind of contact you have with the care home / care home staff? Are you only there because of Ms./Mr. (name) or do you look after other patients there?<br>- For home visits<br>- Accompaniment to practice appointments<br>- Telephone contact<br>- When, how often; what if a resident falls ill in between?
What topics do you talk about with the care home / care home staff?
How would you describe the usual procedure of this care home with regard to hospital admissions?
Please describe your relationship with Ms./Mr. (name). In your opinion, what kind of patient is Ms./Mr. (name)?
Tell us how you assess Ms./Mr. (name)'s physical and mental condition. How mobile is Ms./Mr. (name)? What illnesses does Ms./Mr. (name) have?<br>How was Ms./Mr. (name)'s condition before the last hospital stay?
Does the care home have an emergency plan in case Ms./Mr. (name) becomes ill? What does this emergency plan look like?

What arrangements have been made with the nursing staff regarding possible deterioration in Ms./Mr. (name)'s condition?
Ms./Mr. (name) was hospitalized unplanned some time ago. Please describe in detail how Ms./Mr. (name) was feeling before the unplanned hospitalisation. How did Ms./Mr. (name) end up in hospital at that time?
What preceded the hospitalisation?
Was the admission on your initiative, by another doctor, by the care home, as an emergency or for any other reason? Why?
How was Ms./Mr. (name) after the hospital stay?
How do you assess the medical necessity of the hospitalisation (s)?
How do you think the hospital stay could have been avoided?<br>Could something have been done differently in advance? / Should something have been done differently to prevent the hospitalisation?
In your opinion, what is missing in the care of Ms./Mr. (name)?
What skills should nursing staff have in order to avoid hospitalisation in such or similar cases?
Would you like to add anything else? Have we covered everything important?

1.2 Nurses

Table 2 Topic guide for nurses

Hello. I am (name) and I work at (institute). We are meeting today because we want to talk about Ms./Mr. (name). You work in this care home and know Ms./Mr. (name). Ms./Mr. (name) was recently in hospital unplanned. I would like to talk to you about your day-to-day work with Ms./Mr. (name) and what it was like when she/he came into hospital.<br>All information you provide will of course be treated confidentially. Everything I learn during our conversation will remain between us and will only be used pseudonymously. This means that your name will not be published together with what you have said.<br>It is also important that there are no wrong or right answers here: I am interested in your opinion and perception. So please tell me everything that is on your mind.<br>If you agree, I would like to record the conversation on tape as previously discussed. If possible, please switch your cell phone / ward telephone to silent mode so that we are not disturbed.
You work in this care home. Tell us something about Ms./Mr. (name) and what a typical working day with her/him looks like.
How long have you been involved in Ms./Mr. (name)'s care? How regularly do you have (nursing) contact with Ms./Mr. (name)?<br>Are there any peculiarities in the daily care?
How are doctor's appointments arranged and carried out?
Ms./Mr. (name) is also cared for by a general practitioner (GP). What kind of contact is there between you and this GP?<br>When and how often does the GP come? Does Dr. (name) make house calls to Ms./Mr. (name)?<br>What happens if a resident needs to see a doctor between scheduled visits?<br>How can you reach Dr. (name) in an emergency?
What topics do you discuss with the general practitioner?
Does the GP tend to make house calls for Ms./Mr. (name) or does Ms./Mr. (name) go to the practice for appointments?
Tell us how Ms./Mr. (name) is doing physically and mentally.<br>How was Ms./Mr. (name)'s condition before the last hospital stay?
Ms./Mr. (name) was unplanned in hospital some time ago. How did it come about that Ms./Mr. (name) ended up in hospital so unplanned?
What preceded the hospitalisation?<br>(Care, contact with family doctor, medical on-call service?)

On whose initiative was the referral made? Why?
Please describe Ms./Mr. (name)'s condition after hospitalisation (after returning to the care home).<br><br>How did you perceive Ms./Mr. (name)'s condition after being admitted to hospital? Was it good or bad that Ms./Mr. (name) was in hospital?
In retrospect, would you say that the hospital stay was necessary? Why?<br><br>Would you admit Ms./Mr. (name) to hospital again in the same situation? If no, why not?
In your opinion, how could the hospital stay have been avoided? What should you have known? What skills/authorizations would have helped you to prevent hospitalisation?
How do you normally deal with deteriorations in Ms./Mr. (name)'s condition or illnesses?<br><br>In which situations do you decide to call a doctor?
In your opinion, what is missing in the care of Ms./Mr. (name)? What could be improved here in the care home?
Would you like to add anything else on the subject of hospital stays? Have we discussed everything important?

1.3 Nursing manager

Table 3 Topic guide for nursing managers

Hello. I'm (name) and I work at (institute). We are meeting today because, as part of the Expand Care project, we want to have a general discussion about the organisation of care in this care home and your work as a care manager. I would like to talk to you about what your day-to-day work looks like, what potential for change you see in the processes here and what changes you may have already implemented in recent years.<br>All information you provide will of course be treated confidentially. Everything I learn during our conversation will remain between us and will only be used pseudonymously. This means that your name will not be published together with what you have said.<br>It is also important that there are no wrong or right answers here: I am interested in your opinion and perception. So please tell me everything that is on your mind.<br>If you agree, I would like to record the conversation on tape as previously discussed. If possible, please switch your cell phone / ward telephone to silent mode so that we are not disturbed.
You've been working at this care home for a very long time as a nursing service manager. Please tell me about your day-to-day work and your scope of practice.
Please tell me what it was like when you started here. What further training have you done since then? How did it come about that you took on the role of the nursing service manager?
What needs for change have you seen here during your time as a nursing service manager?<br>(e.g. in relation to care processes, duty planning, internal and external communication, further training, etc.)
What was better in the past, what was worse? Are there processes that have changed significantly in recent years?
What changes have you been able to implement over time?
What changes would you have liked to implement but were unable to do so?
What other changes would you like to see?
How do you rate interprofessional communication (e.g. with GPs and specialists) with regard to the residents?
Could you please tell me a bit about how cooperation and communication with the residents' relatives work?
Please tell me how communication with the control center, the emergency doctor and the rescue service normally takes place in emergencies.
What is it like to communicate with hospitals? For example, when someone is about to be admitted or discharged?

What role does “Advance Care Planning” play in your work with residents?
What role do health promotion and prevention activities play in everyday care?
How is your team usually made up? Which professions or qualifications are represented?
What skills do you lack in the team? What skills would you still like to have yourself?
Which (health) care needs of your residents would you like to serve even better? What would be necessary for this?
What needs to change in order to further improve the care of your residents?
What would you like to see more time or better remuneration for?
Would you like to add anything to the topics we have just discussed? Have we covered everything important?

1.4 Relatives and significant others

Table 4 Topic guide for relatives

Hello, I am (name) and I work at (institute). We are meeting today because we are interested in your relative's unplanned stay in hospital. This means that I would like to talk to you about what it was like when she/he was hospitalized.<br><br>All information you provide will, of course, be treated confidentially. Everything I learn during our conversation will remain confidential and will only be used pseudonymously. This means that your name will not be published together with what you have said and neither the general practitioner nor the health insurance company or the care home will get to know what you have told me during this conversation.<br><br>It is also important that there are no wrong or right answers here: I am interested in your opinion and perception. So please tell me everything that is on your mind.<br><br>If you agree, I would like to record the conversation on tape as previously discussed. If possible, please switch your cell phone to silent mode so that we are not disturbed.
Ms./Mr. (name) lives in a care home. Tell us what you typically have to do for and with Ms./Mr. (name).
Ms./Mr. (name) was in hospital some time ago. Tell me how Ms./Mr. (name) was before the hospital stay.<br><br>How was Ms./Mr. (name)'s condition before the last hospital stay?
How did it come about that Ms./Mr. (name) ended up in hospital?
Do you talk to the nursing staff about Ms./Mr. (name)'s state of health? What does this typically look like? Would you please describe this for the time before hospitalisation?
What preceded the hospitalisation? What happened before?
Were there any peculiarities of Ms./Mr. (name)'s care situation? Which ones?<br><br>Were there any peculiarities in the general practitioner's care? Which ones?
Was the referral made by the general practitioner/ a specialist, as an emergency, at your request or for another reason? Why?
How was Ms./Mr. (name) after the hospital stay?
What do you usually do when you notice that Ms./Mr. (name) is feeling worse?<br><br>Who do you talk to about it?<br><br>What happens when Ms./Mr. (name) is feeling worse?
What else would you wish for the care and support of Ms./Mr. (name)?

What else would the nursing staff need to be able/allowed to do so that you could take better care of Ms./Mr. (name)?
In your opinion, was Ms./Mr. (name)'s hospitalisation necessary?<br>In your opinion, how could the hospitalisation have been prevented?
Would you like to add anything else on the subject of hospitalisation? Have we discussed everything important?

1.5 Residents

Table 5 Topic guide for residents

Hello. I am (name) and I work at (institute). We are meeting today because I am interested in your unplanned hospitalisation. In other words, I would like to talk to you about what it was like when you were admitted to hospital unplanned.<br><br>All information you provide will of course be treated confidentially. Everything I learn during our conversation will remain confidential and will only be used pseudonymously. This means that your name will not be published together with what you have said and neither your general practitioner nor the health insurance company or the care home will find out what you have told me in this conversation.<br><br>It is also important that there are no wrong or right answers here: I am interested in your opinion and perception. So please tell me everything that is on your mind.<br><br>If you agree, I would like to record the conversation as previously discussed. If possible, please switch your cell phone to silent mode so that we are not disturbed.<br><br>Do you have any questions before we start?
You live in a care home. Tell me what the nursing staff do for and with you on a typical day.
You had an unplanned stay in hospital some time ago.<br><br>Please tell me how your health was before your unplanned stay in hospital?
How did it come about that you ended up in hospital?
What happened before the hospitalisation?
Were there any peculiarities regarding your state of health and your nursing/medical care?
Do you sometimes talk to the nursing staff about your state of health? How does such a conversation typically take place? Would you please describe this for the time before you were admitted to hospital?
Who arranged for you to be hospitalized? (Was it your general practitioner or a specialist, the nurses here, yourself or your relatives?)<br><br>Was it an emergency or was there another reason? Why?
How did you feel after the hospital stay?
What do you usually do when you realize that you are not feeling well?<br><br>Who do you talk to / what do you do?
What else would you wish for your care and support?<br><br>What else should the nursing staff be able/allowed to do so that they can take even better care of you?

In your opinion, was the hospitalisation necessary? Why?<br>In your opinion, was there another possibility/alternative in the situation?<br>In your opinion, how could the hospitalisation have been avoided?
Would you like to add anything else on the subject of hospital admissions or stays? Have we discussed everything important?

Section 2 – Supplementary material to results

1. Case summaries

To veil participants' gender, we use the pronouns "they/their/them" instead of "he/his/him" or "she/her".

Case 1

Resident living with dementia and repeated falls

The key event in this case was hospitalisation after the resident fell and acquired a minor head wound. The resident ("they" or R1 in the following) lived with dementia, showed a wandering behaviour with a history of repeated falls and fall related injuries. R1 had received antipsychotic medication since entry into the facility several years ago. After an initial period of higher dosage, medication had been reduced. R1 was provided with a helmet and hip protectors to prevent fall-related injuries but accepted these only intermittently. Additionally, R1 had a wristband alerting staff if they left the facility. Their spouse visited regularly and communicated with nurses in charge and the general practitioner (GP). The spouse initiated a medication review with the GP after observing R1 to appear dizzy. Regarding the decision to transfer R1 to the hospital after the fall, nursing staff referred to a general routine to hospitalise any resident after a fall if injuries to the head occurred. Overall, R1's spouse was very satisfied with the care provided by staff and felt that they reacted well if they suggested improvements for their spouse's care.

Case 2

Resident living with anaemia and advance directive

The key event in this case was hospitalisation with severe anaemia. The resident (R2) experienced repeated upper gastrointestinal bleeding. R2 repeatedly refused hospitalisation and had an advance directive documenting this wish. When their condition worsened, the GP and R2's adult child had a conversation with R2 about the life-threatening situation and R2 consented to hospitalisation. According to the nurse in charge R2 had decided after this conversation that they were not prepared to die after all and therefore revised their former decision.

Case 3

Resident living with unclear health deterioration and cancer

The key event was a hospitalisation after the resident experienced unclear health deterioration. The resident (R3) had occasional falls due to vertigo, further symptoms were nausea and obstipation. Nursing staff observed R3 had a reduced vigilance and attributed this to belated symptoms of a head injury after a fall and therefore admitted R3 to the hospital. Diagnostic showed a life-threatening atypical sepsis which was successfully treated. During diagnostics, the incidental finding of a malign tumour occurred, and the hospital physician recommended palliative treatment because of R3's older age. The GP decided together with R3's adult child to follow this recommendation, while R3 was not informed about the diagnosis. R3 had an advance directive stating that they did not wish life-prolonging antibiotic treatment, which the GP was not informed of. R3 mentioned a constant feeling of insecurity because they felt not informed about their medical treatment but did not dare to speak

with nursing staff or doctors as R3 felt these carers did not have enough time, R3 wouldn’t understand their explanations and R3 did not want to cause trouble.

Case 4

Resident living with obesity, impaired mobility and heart failure

The key events were resident’s repeated ambulance transports and hospitalisations for minor surgery of the peripheral infection of a limb. Resident’s (R4) obesity as well as prescription of anticoagulants required special care arrangements which were repeatedly not communicated or implemented. Previously, R4 had experienced two heart attacks with subsequent emergency treatment and hospitalisation. The nurse in charge and the nurse assistant remembered a very sudden change (shortness of breath) in resident’s health status only shortly before the heart attack and commented on how fast R4 deteriorated. An adult child of R4 reported having observed swollen hands and dark urine already in the days preceding the second heart attack. They would contact the GP timely if observing this again to ensure the information was adequately transferred. In general, R4 felt lonely in their room but was not often transferred in a wheelchair and brought into communal areas. The nurse justified this with the top priority to prevent pressure ulcers. These could be caused by the sling that had to remain in the wheelchair when using the patient lift. R4 would be too heavy to be transferred by two nurses without a patient lift. Nutrition was a topic of discussion between nursing staff and R4’s adult child but the diet was not changed consequently. They hesitated to deny R4 sweets and cookies as these were one of few joys in R4’s life and they wanted R4 to decide on their own about this. Overall, apart from lack of mobilisation, the adult child was very satisfied with the care provided in the care home.

Case 5

Resident living with dementia and repeated falls

The key event in this case was a hospitalisation after a fall. It was the fourth time in eight weeks that the resident (R5) fell and was hospitalised. The resident lived with dementia and regularly showed aggression towards care home staff. R5 had antipsychotic medication prescribed. Falls were viewed as unavoidable by care home staff and R5’s adult child and prevention measures focused on avoidance of tripping hazards. Nursing staff based their decisions for hospitalisation on symptoms but also on implicit rules (“head wounds always lead to hospitalisation”) and fear of liability. Hospital stays worsened R5’s condition and their challenging behaviour substantially and hospital staff contacted the care home for advice to handle R5. A nurse assistant described that R5 was quite nice if one treated them with kindness and knew their ways and preferences.

2. Case-based results: causal factors, generalising questions and fields of action

Table 6 Causal factors, questions and fields of action case 1

Case	Causal factors	Questions	Fields of action
**1**	• Relatives initiated medication review instead of nurses<br>• Not a nurse, but a relative observed dizziness and interpreted this as related to antipsychotic medication<br>• Medical treatment was not adjusted after key events<br>• Nurses took measures to prevent fall related injuries but not falls<br>• Nurse communicated with GP and involved specialist after relative's request.<br>• Nurses followed seemingly implicit rules on how to decide on hospitalisation, apart from the assessment of resident's actual condition	• What is the role of nurses in the management of neuroleptic / antipsychotic medication?	• **Nursing support in the therapy of chronic diseases**<br>• *Evaluation of the care situation*<br>• *Quality of care based on current scientific standards*
		• Why does the relative initiate a review of neuroleptics/antipsychotic medication with the GP and not the nurse?	• *Symptom control in chronic diseases*<br>• *Reaction to nursing assessments*<br>• **Participation of relatives**<br>• *Relatives as a source in care processes*
		• What do nurses understand by prophylaxis/preventive actions regarding falls in long-term care?	• **Active prevention**<br>• *Management of medical and care devices*
		• How does communication between GPs and specialists take place regarding residents in long-term care and which role do other involved parties play?	• **Communication with general practitioners and specialists**<br>• *Multiprofessional checklist*<br>• *Maintaining a trans sectoral care network*
		• Do values, implicit and explicit facility rules lead to more hospital admissions than necessary?<br>• What is the role of previous failure/success in critical situations in care in decision-making processes?<br>• Which explicit or implicit standards exist for information flow, scope of practice and decision-making in long-term care?	• **Handling rules and protocols**<br>• **Shaping learning processes in the facility**<br>• **Organising teams with mixed skills levels**<br>• *Communication in teams with mixed skills levels in the facility*

Fields of action in bold: first found in this case. Fields of action in italics: applied to case after developed in other cases.

Table 7 Causal factors, questions and fields of action case 2

Case	Causal factors	Questions	Fields of action
**2**	• Resident (R2) refused hospitalization and had an advance directive.<br>• When their condition worsened, the GP was informed and explained R2 would die if they didn't go to the hospital, so they consented to admission.<br>• Resident felt they could not talk to or understand information from nursing staff and R2's GP did not visit.<br>• Nursing assistants were not included in hand overs, had to excerpt information from residents' electronic records.	• Are nurses aware of legal requirements and differences in documents like advance directives and living wills?<br>• Why is palliative care not organised for a resident who has an advance directive and refuses hospital, but instead the resident convinced to consent to hospitalisation by the GP?<br>• How is palliative care organised in care homes?	• *Evaluation of the care situation*<br>• *Recognising and considering concerns of Residents regarding hospitals*<br>• *Prioritisation of care needs*<br>• *Enabling empowerment and participation*<br>• *Advance Care Planning*<br>• *Nurses as advocates of residents*
		• How are visits and rounds by GPs or specialists organised and coordinated within long-term care facility?<br>• Why is medical information not communicated to residents in an age- or ability- appropriate manner?	• *Communication with relatives and residents*<br>• *Communication with general practitioners and specialists*
		• How effective are communication processes between nurses and nursing assistants regarding changes in residents' health?	• *Communication in teams with mixed skills levels in the facility*<br>• *Organising teams with mixed skills levels*

Fields of action in bold: first found in this case. Fields of action in italics: applied to case after developed in other cases.

Table 8 Causal factors, questions and fields of action case 3

Case	Causal factors	Questions	Fields of action
**3**	• Nurses contacted the GP but gave only unspecific information by telefax (worsening condition).<br>• Unspecific signs of infection were not recognized timely.<br>• Resident was admitted to hospital and treated with antibiotics although an advance directive was in place that stated otherwise.<br>• The GP was not informed about the advance directive.<br>• Resident was not informed about cancer diagnosis.<br>• Resident does not understand their medication.<br>• Resident felt lonely and isolated since they first moved into the facility.<br>• Resident feels that neither nursing staff nor GP have time to talk to them.<br>• Repeated falls were not systematically evaluated for their reasons, it depends on the experience of the nurse whether this is addressed.<br>• The relative assumes that the resident does not receive appropriate care because the approved level of care does not provide enough financial reimbursement.	• How well do nurses recognize early symptoms of deterioration?	• *Symptom control in chronic diseases*
		• How can changes in prescriptions at resident's return from hospital be addressed to ensure timely care in long-term care facilities?	• **Medication after hospitalisation**
		• Are nurses aware of legal requirements and differences in documents like advance directives and living wills?<br>• What is the understanding of autonomy of residents by those involved in care?	• **Advance Care Planning**
		• How are contacts and communication processes with specialists organised in long-term care facilities?<br>• How is communication about difficult diagnoses organised in long-term care facilities?<br>• How should nurses address health issues with residents which are not addressed or avoided to be communicated by general practitioners or relatives?<br>• Are residents encouraged to communicate about their health or care needs in long-term care facilities?	• **Maintaining a trans-sectoral care network**<br>• **Nurses as advocates of residents**<br>• *Participation of relatives*<br>• *Communication with relatives and residents*<br>• *Communication with general practitioners and specialists*
		• How are processes for assessing and adjusting resident's care level managed in long-term care facilities?<br>• When do nurses act proactively regarding nursing interventions for residents?<br>• What role do relatives play in initiating medical measures for residents?<br>• How can residents be socially integrated when first moving into a long-term care facility from their own homes?	• **Management of residents' approved care level**<br>• **Management of medical and care devices**<br>• **Psychosocial management of residents moving into the facility**<br>• *Evaluation of the care situation*<br>• *Reaction to nursing assessments*

Fields of action in bold: first found in this case. Fields of action in italics: applied to case after developed in other cases.

Table 9 Causal factors, questions and fields of action case 4

Case	Causal factors	Questions	Fields of action
**4**	• Nurses refrain from moving resident into a wheelchair because they see danger of pressure ulcers in this position.<br>• Nurses were surprised by heart attacks whereas relative noted edema, shortening of breath and dark urine.<br>• Several appointments in hospital for routine surgery without result because of wrong ambulance care, belated transport, medication not paused.<br>• Resident feels lonely, ambulation and social contacts more important than intact skin.<br>• Resident is heavily overweight which is a major cause for their health problems.<br>• Neither professionals nor relatives do consequently support weight loss.<br>• Communication flow between resident, relatives, nurses and GP is incomplete.<br>• Resident talks only to relative, relative talks to GP, nurses receive information from GP.<br>• Several long-term conditions (hearing aids, impaired eye sight, repeated peripheral infections) are	• How well do nursing staff know the national standard for the care of chronic wounds and what knowledge do they have about pressure ulcers?	• **Quality of care based on current scientific standards**<br>• *Nursing support in the therapy of chronic diseases*
		• Are nurses able to recognise symptoms of cardiac decompensation?	• **Symptom control in chronic diseases**
		• Do nurses know the fields of action of different medical service providers and how services can be financed?<br>• What role do nurses play in management of medication changes before residents go into surgery outside of the long-term care facility?<br>• What are the communication processes between the long-term care facility, the emergency centre and hospitals?<br>• Can other health care professionals such as physiotherapists be integrated into care planning and seen as a resource for a nursing-related health problem?<br>• Why are residents afraid of going to the hospital?	• **Case-management**<br>• **Multi-professional checklist**<br>• **Nurses competence for prescription of defined medical services**<br>• **Recognising and considering concerns of residents regarding hospitals**
		• How are care problems prioritised when the resident's or relative's assessment of urgency differ?<br>• How are discrepancies in the results of nursing assessments identified and a comprehensive assessment established?	• **Prioritisation of care needs**<br>• **Enabling empowerment and participation**
		• What are the consequences for the continuity of care of resident's poor diet and weight?<br>• Do nurses support lifestyle changes in case this leads to health problems?	• **Strategies for health promotion**
		• How do those involved in care communicate about the residents' state of health?<br>• Why are there barriers in communication about resident's well-being between nurses and relatives?	• **Participation of relatives**<br>• **Relatives as a resource in care processes**<br>• *Nurses as advocates of residents*<br>• *Communication with relatives and residents*

	not monitored and addressed by nursing staff.<br>• Service provision like visiting specialists is impeded by residents' overweight and lack of mobility.<br>• Care homes monitor residents' weight closely but address only weight loss.	• To what extent do care professionals take the residents' social network into account in the management of care?	
		• Are care processes of residents evaluated and adjusted after critical health events?<br>• Was the tendency for repeated peripheral infection recognised?<br>• Are nurses adequately able to evaluate complex information, recognise correlations, derive needs for action and initiate comprehensive measures?<br>•	• **Involvement of nurses in medical care**<br>• **Understanding health as a life course process**<br>• **Evaluation of the care situation**<br>• **Reaction to nursing assessments**<br>• *Management of medical and care devices*
		• Are nurses aware of explicit and implicit values in decision-making processes within long-term care?	• *Handling rules and protocols*<br>• *Protocols as guidance*

Fields of action in bold: first found in this case. Fields of action in italics: applied to case after developed in other cases.

Table 10 Causal factors, questions and fields of action case 5

Case	Causal factors	Questions	Fields of action
**5**	• Hospital staff are unable to cope with resident's challenging behavior and send her back to the care home.<br>• Continuity of care in transitions (e.g. medication provision) is disrupted and leads to health deterioration and complications.<br>• Emergency services do not consider special needs of people with dementia.<br>• A case conference was conducted but on resident's weight loss and not about her repeated falls and antipsychotic medication.<br>• This was discussed with the GP, but medication was not adjusted.<br>• Decisions about hospital admissions are guided by fear of litigation or "to be on the safe side".	• How can timely care be provided for residents who have been discharged from hospital?<br>• Are special needs of cognitively impaired residents communicated in between health care sectors?<br>• How are people with dementia integrated into care settings?<br>• How can care professionals ensure that care in hospital is more individualised for people with dementia coming from long-term care facilities?	• *Case management*<br>• *Multiprofessional checklist*<br>• *Nurses competence for prescription of defined medical services*<br>• *Recognizing and considering concerns of residents regarding hospitals*<br>• *Maintaining a trans sectoral care network*<br>• *Medication after hospitalisation*
		• How are topics for case reviews selected and prioritised in long-term care facilities?<br>• When do nurses initiate case conferences or case reviews in long-term care facility?<br>• How are longer-term processes and developments considered in care planning?	• *Case management*<br>• *Evaluation of the care situation*<br>• *Understanding health as a life cause process*<br>• *Reaction to nursing assessments*<br>• *Nursing support in the therapy of chronic diseases*<br>• *Prioritisation of care needs*<br>• *Communication with relatives and residents*
		• How are the benefits and burdens of hospitalisation considered for residents in decision making?<br>• Is the burden of hospitalisation perceived for the residents?<br>• What role do legal protection and legal consequences play in the decision-making process of hospital admissions?	• *Handling rules and protocols*<br>• *Standards as guidance*

Fields of action in bold: first found in this case. Fields of action in italics: applied to case after developed in other cases.

3. Fields of action, goals and interventions

Table 11: Fields of action, goals and interventions to eliminate gaps and breaks in care

Meta-theme	Field of action	Goal	Nursing activities / interventions
**1. Geriatric nursing care**	1.1. Symptom control in chronic diseases	Exacerbation of chronic diseases is detected early.	• Selection of suitable assessment tools for chronic conditions<br>• Implementation of (in-house) standards (checklist or existing instruments) for the assessment of chronic diseases<br>• Ensuring that nursing assessments are carried out<br>• Interpretation of assessment results in terms of need for action and urgency
	1.2. Involvement of nurses in medical care	Timely evaluation and adjustment of medical care in the facility by the practitioner is guaranteed.	• Obtain permission of residents to view communication about health status (doctor's letters, discharge letters)<br>• Clarification with residents what level of involvement in coordination of medical care they desire<br>• Initiation of medication review for defined events and according to clinical assessment
	1.3. Understanding health as a life course process	Nursing care and the initiation of relevant health services are based on health and nursing anamnesis and the results of regular nursing rounds.	• Regularly evaluate the state of health comprehensively on the basis of defined criteria and including resident's biography<br>• Derive recommendations from resident's health status and nursing rounds and initiate interventions beyond the service provided by the facility
	1.4. Case-management	Potential health-promoting care services by other health care professions are identified and initiated in a timely manner.	• Regular review of health care provision<br>• Communication with other care providers<br>• Initiation of multi-professional interaction with care providers
	1.5. Evaluation of the care situation	The care situation is regularly evaluated and adjusted if necessary.	• Evaluation of care situation through regular nursing rounds and inclusion of the results of previous evaluation results<br>• Establish new goals if necessary and derive appropriate interventions<br>• Document results of nursing rounds in care plans accessible to other providers

			• Regular discussions with other care providers on the status and further course of care
	1.6. Reaction to nursing assessments	Timely reactions to changes in the state of health are guaranteed.	• Selection and implementation of resident-related assessments<br>• Check existing assessments for timeliness and indication<br>• Review resident's health status using appropriate assessments<br>• Nursing, medical or other services of the health care system are initiated on the basis of the assessment
	1.7. Quality of care based on current scientific standards	Nursing care fulfils current scientific standards.	• Ensure availability of the expert standards and guidelines<br>• Review current care processes and pathways<br>• Adapt standards and guidelines to the context of the facility and implement and evaluate them<br>• Carry out training on expert standards for nurses in the facility<br>• Support and offer nurses training (peer-to-peer)
	1.8. Nursing support in the therapy of chronic diseases	Side effects of frequently used medication groups are recognised and appropriate measures are initiated.	• Make information available on common medicines with common side effects<br>• Formulate recommendation for targeted relative education and subsequent communication with physicians about medication reviews<br>• Targeted monitoring of medication changes<br>• Establish information material and training for all staff
		All sources of information about resident's health concerns are considered. The organisation of medical care is coordinated individually.	• Ensure structured communication with relatives about the distribution of tasks and responsibilities in medical care
**2. Interprofessional collaboration**	2.1. Multi-professional checklist	Important aspects relevant to transport and procedures such as surgeries are complied with.	• Inform other care providers about relevant health aspects<br>• Implementation of a multi-professional and trans-sectoral checklist for residents
	2.2. Nurses competence for prescription of defined medical services	Interventions for medical care are prescribed promptly.	Prescription of e.g.:<br>• Residents' transport to other services<br>• follow up medication initially prescribed by general practitioners

			• therapeutic measures
	2.3. Recognising and considering concerns of residents regarding hospitals	Causes of residents' anxiety and concerns about hospital transfers are clear and communicated to all providers involved.	• Clarify concerns through discussion with residents<br>• Treat anxiety and concerns with empathy.<br>• Communicate residents' concerns at admission to hospital
	2.4. Maintaining a trans-sectoral care network	Timely (specialist) medical care for residents is ensured.	• Reserve time slots with medical clinics for residents<br>• Agree on structured communication pathways with medical clinics, e.g. via urgency levels<br>• Initiate case conferences by telephone or video with residents and physicians for complex cases
	2.5. Medication after hospitalisation	Medication prescribed by medical staff are provided to residents within a reasonable period of time.	Since prescription of follow-up medication by hospital clinicians is possible:<br>• Advocate for patient's entitlements in the context of discharge and request prescriptions from the hospital<br>• establish information about the possibility of prescriptions in hospital discharge management in the facility
**3. Empowerment of residents**	3.1. Prioritisation of care needs	Care problems are prioritised according to residents' needs as well as the quality of care.	• Initiate nursing visits and case discussions involving relatives or confidants regularly<br>• Resolve conflicting assessments of care problems with resident preferences
	3.2. Enabling empowerment and participation	Residents are empowered to make informed decisions.	• Residents are informed about the results of nursing assessments, benefits and risks of interventions and are empowered to make an informed decision<br>• Consider residents' wishes even if they collide with measures seen as appropriate to secure quality of care<br>• Initiate nursing visits and case discussions involving relatives or confidants regularly
	3.3. Advance Care Planning	Residents' wishes are considered in the provision of nursing and medical care and are known to all those involved in their care.	• Up-to-date knowledge of advance care documents and proxy decision-making are made available to all care providers<br>• Establish a common understanding and procedures for dealing with advance care planning at the facility level

**4. Health promotion and prevention**	4.1. Strategies for health promotion	Residents are informed about opportunities to take health-promoting measures and are supported in formulating health goals.	• Determine individual health values and realistic goals in a structured dialogue with the resident<br>• Identify and plan measures to support individual health behaviour and adaptation of the environment and guide implementation.
	4.2. Active prevention	Processes leading to adverse events are understood and identified and preventive measures initiated without delay.	• Regular case conferences<br>• Carry out root cause analysis after critical events
**5. Communication processes**	5.1. Participation of relatives	Establish trusting communication with relatives so that information is not only passed on but a joint discussion and assessment is developed.	• Conduct case conferences in which relatives are included<br>• Actively seek the assessment of relatives as part of care planning and the care process
	5.2. Relatives as a resource in care processes	Relatives are considered in the care process.	• Check for social resources of residents<br>• analysis of potential support network for residents<br>• Initiation of communication with social network<br>• Use social network as a source of information on needs in regular meetings<br>• Maintain and document contacts within the social network
	5.3. Nurses as advocates of residents	Nurses see themselves as advocates for the resident and promote their autonomy.	• Encourage residents to communicate<br>• Involve all involved parties in communication processes<br>• Design communication as a trialogue<br>• Ensure age- und illness appropriate communication with residents
	5.4. Communication with relatives and residents	Residents and relatives know who is caring for them, what their function is and what they are responsible for so that information about health concerns reaches the right people.	• Legible name badges are worn by staff<br>• Up-to-date pictures and overview of staff in facility<br>• Laminated overview of staff per ward in residents' rooms<br>• Overview of "most important" contact persons for relatives incl. responsibilities
	5.5. Communication with general practitioners and specialists	All care providers are informed about current diagnoses and therapies, all involved specialists and physicians receive consistent and up to date	• Use electronic resident file for the exchange of information<br>• Implement interprofessional documentation within the facility for all health professionals involved

		information about the state of health of residents.	• Implement forms and documents for structured communication<br>• Structured information transfer via SBAR
	5.6. Communication in teams with mixed skills levels in the facility	Scope of practice, responsibilities and information channels of the different professions and hierarchical levels are known.	Organisation of communication in skill-mix in facility via:<br>• Structured reflection and communication about teamwork with the team<br>• Implementation of a decision flow chart<br>• Structured dissemination of information via SBAR
**6. Management**	6.1. Management of residents' approved care level	Care needs are adequately refinanced and staffing ratios is calculated appropriately.	• Determine changed need for care and initiate a review of care level<br>• Inform relatives about the fact that a request for a review of care needs is being made
	6.2. Management of medical and care devices	Care devices are provided according to residents' needs.	• Review the need for medical and care devices.<br>• Formulate recommendations for prescription of devices<br>• Initiate provisions and prescription for devices
	6.3. Psychosocial management of residents moving into the facility	Social needs of resident moving into facility are considered and move-ins are individually tailored to needs.	• Arrangement of admission taking (psycho)social aspects into account<br>• Observation of the psychosocial condition in the first days after moving in
**7. Values, standards and guidelines**	7.1. Handling rules and protocols	Balanced use of protocols (quality assurance of processes versus individual decision-making)	• Carry out an inventory of existing implicit and explicit rules and protocols in facility<br>• Develop criteria to measure the outcomes of protocols<br>• Regularly evaluate the use of protocols<br>• Update protocols based on evidence
	7.2. Protocols as guidance for decision-making	Protocols support decision-making on hospital admissions	• Implement decision-making aids for situations that result in hospital admission (e.g. falls, dehydration)
	7.3. Shaping learning processes in facility	Use experiences with successes and failures for the further development of professional and personal competence	Reflection on decision-making in the context of:<br>• Structured case discussions<br>• Collegial counselling

			• Supervision
	7.4. Organising teams with mixed skills levels	Scope of practice, responsibilities and information channels of the different professions and hierarchical levels are known	Organisation of skill-mix in facility via:<br>• Job descriptions<br>• Familiarisation concepts<br>• Structured reflection and communication about teamwork with the team<br>• Implementation of a decision flow chart<br>• Designation of responsible persons for support in the respective process<br>• Structured dissemination of information via SBAR
